# Heteromeric amyloid filaments of TDP‐43 and annexin A11 in neurodegenerative diseases

**DOI:** 10.1002/alz70855_104408

**Published:** 2025-12-24

**Authors:** Benjamin Falcon

**Affiliations:** ^1^ MRC Laboratory of Molecular Biology, Cambridge, United Kingdom

## Abstract

**Background:**

Neurodegenerative diseases are characterised by the accumulation of amyloid filaments of specific proteins in the central nervous system. Human genetic studies have established a causal role for amyloid filaments in neurodegeneration. However, the underlying molecular mechanisms remain poorly understood, which limits progress in targeting amyloid filaments for diagnostic and therapeutic benefit.

**Method:**

We used electron cryo‐microscopy (cryo‐EM) to determine the atomic structures of amyloid filaments from the brains of individuals with neurodegenerative diseases.

**Result:**

Unexpectedly, this approach directly revealed that TAR DNA‐binding protein 43 (TDP‐43) co‐assembles with a second protein, annexin A11 (ANXA11), to form heteromeric amyloid filaments in specific neurodegenerative diseases. The atomic structures of these heteromeric filaments guide mechanistic studies into their formation.

**Conclusion:**

This work establishes a central role for ANXA11 in neurodegenerative diseases. The unprecedented formation of heteromeric amyloid filaments in the human brain revises our understanding of amyloid assembly and may be of significance for the pathogenesis of neurodegenerative diseases.

